# Supplementary material for: Somatic hypermutation of T cell receptor α chain contributes to selection in nurse shark thymus
Source: eLife. 2018 Apr 17;7:e28477. doi: 10.7554/eLife.28477 (PMC5931798; doi:10.7554/eLife.28477)
Supplement: Figure 4—source data 1. — Alignment of nucleotides belonging to the join between variable (V) and joining (J) segments within TcRα thymocyte clones. We determined the putative ends of each V segment and putative beginning of each J segment by comparing alignments between different sharks, assuming that identical nucleotides between sharks were germline. The last number of each sequence name indicates the number of clones containing that nucleotide sequence between the V and J segments. [file elife-28477-fig4-data1.docx]

-------------------------------10--------20--------30---

----------------------....|....|....|....|....|....|....

aV1J_T17_100710_2-----TGT-------------------------------

aV1m_T14_080912_2-----TCCGCTTACG------------------------

aV1m_T21_072012_2-----CCCGTAT---------------------------

aV1J_V14_101310_2-----CCCCCATATACT----------------------

aV1m_T17_071212_2-----CCGGACT---------------------------

aV1m_T13_072610_1-----CCGCGCT---------------------------

aV1J_V21_101310_1-----CA--------------------------------

aV1J_V09_110210_1-----TCC-------------------------------

aV1J_V19_110210_1-----CCGG------------------------------

aV1J_V07_091410_1-----TCCCCT----------------------------

aV1J_V05_091010_1-----CCGGTA----------------------------

aV1m_T08_060512_1-----TCGGGA----------------------------

aV1m_T13_060512_1-----CCAAAAAAAT------------------------

aV1J_V08_102810_1-----GCGCTGTGG-------------------------

aV1J_V20_110210_1-----CTCCTGCGAT------------------------

aV1J_V02_110210_1-----A---------------------------------

aV1m_T02_060512_1-----CCGCCGCAT-------------------------

aV1B_A_419.40_1-------CAACA-----------------------------

aV1m_T14_060512_1-----ACCTTACT--------------------------

aV1J_V24_101310_1-----CGGGGCCACTACGGAACTGGTACT----------

aV1J_V05_102810_1-----CACGGG----------------------------

aV1J_V05_101310_1-----CCGGGA----------------------------

aV1.4J_V07_090110_1---GCCGGGTACT------------------------

aV1.4J_V08_101310_1---GCCGGGTACTGGAATA------------------

aV1.4J_V17_090110_1---GCCGGGTACTGGAATAACT---------------

aV1.3J_V01_091410_2---CCTATTGGGGGTTACAGC----------------

aV1.2J_V15_091010_2---CCACGGG---------------------------

aV1.2J_V23_090110_1---CCACGG----------------------------

aV1.2J_V18_110210_1---CACGG-----------------------------

aV1.1J_V18_101310_1---CGGGGCCACTACGGAACTGGTACTGG--------

aV1.1J_V08_110210_1---CGGGGCCACTATGGAACTGGTACT----------

aV1.1J_V01_101310_1---CGGGGCCACTACGGAGATGAGACT----------

aV2.1m_T22_071212_5---TTCGCCGGTAT-----------------------

aV2.1J_V06_091410_1---GAT-------------------------------

aV2.1J_T43_100410_1---CCCC------------------------------

aV2.1B_A419.3---------TGGCC-----------------------------

aV2.2m_T13_080212_1---TTTGAGAAAGAAT---------------------

aV2.2m_T04_071212_2---CGGACTGGAGGGTTTACA----------------

aV2.2J_V22_110210_1---TACCCTGGATTCTCTGAACAA-------------

aV2.3m_T11_070612_2---CCGGTCGAAT------------------------

aV2.3J_V12_102810_1---CCGACCT---------------------------

aV2.3J_V07_110210_1---CGAGCCGCCGCATGGCTGAT--------------

aV3m_T23_051410_1-----CC--------------------------------

aV3B_A_419.29_1-------CCGTCC----------------------------

aV3m_T32_051410_1-----CCCGAGATA-------------------------

aV3m_T30_051410_1-----TCAATGGGGAGCTGGGACT---------------

aV3m_T20A_060712_1----CTCGGAC---------------------------

aV3J_V24_110210_1-----TCGAGGGGGCGTACACCAATCCTCCAC-------

aV3J_V21_110210_1-----ATGAGGACTGGAGCTTT-----------------

-------------------------------10--------20--------30---

----------------------....|....|....|....|....|....|....

aV3m_T21_051410_1-----TCGGCT----------------------------

aV3m_T29_051410_1-----CCGAAGGAAGGAT---------------------

aV3m_T08A_060712_1----CAGACGAT--------------------------

aV3B_A_419.4_1--------TCCCCTAAG-------------------------

aV3m_T11A_060712_1----ACCCCG----------------------------

aV3m_T10_052410_1-----CTCGTCGAACTTTGGAGGTAACAC----------

aV3m_T16A_060712_1----CTCGAGA---------------------------

aV3m_T24_051410_1-----CTCGATCTCGTCGCTTGC----------------

aV3m_T04A_060712_1----CCAT------------------------------

aV3J_V19_091010_4-----GCG-------------------------------

aV3J_V06_102810_2-----CTCGCTCTCG------------------------

aV3J_T21_100710_2-----TCTAGTTTCT------------------------

aV3J_V16_090110_1-----TCGGGCAGGCTACTCC------------------

aV3J_V15_102810_1-----GACCT-----------------------------

aV3m_T13A_060712_1----CCGGTCCCGCGCTAACGCC---------------

aV3m_T31_051410_1-----TGTACT----------------------------

aV3J_T21_100410_1-----CAA-------------------------------

aV3B_A_419.11_1-------CCAAATACT-------------------------

aV3m_T27_051410_1-----C---------------------------------

aV3m_T23A_060712_1----TTCCACTGATTCAGGGGGTGCCACTCCAATAATA

aV3J_V06_110210_1-----GTCCGGG---------------------------

aV3m_T28_051410_1-----GGAC------------------------------

aV3m_T26_051410_1-----CTCGCGTCAT------------------------

aV3m_T35_051410_1-----T---------------------------------

aV4m_T13_070612_1-----CCCGGATCGGGG----------------------

aV4m_T12_060612_1-----GAACTAC---------------------------

aV4J_V01_102810_1-----CCTAGCGGCTAC----------------------

aV4m_T11_060612_1-----CCGCCC----------------------------

aV4B_A_419.33_1-------CCCCGTGCTGGAGCAGCTTCT-------------

aV4J_T10_100710_1-----GCTTGGGGAT------------------------

aV4m_T12_071612_1-----CGG-------------------------------

aV4m_T09_061812_1-----CACGCGG---------------------------

aV4J_V12_091410_2-----TCG-------------------------------

aV4m_T07_061812_2-----CTGCG-----------------------------

aV4m_T04_062512_2-----CCCGGATATGAAC---------------------

aV4m_T24_080912_1-----TCTCAACCCCCCG---------------------

aV4m_T09_080212_3-----AAAAGCCGGGCAT---------------------

aV4m_T24_071612_3-----CTCCAATGCCCG----------------------

aV4J_V10_110210_2-----CCTAGTGGTT------------------------

aV4m_T04_061812_1-----CCGGACGCCG------------------------

aV4J_V23_110210_1-----CCTAGTGGTTAC----------------------

aV4J_V03_110210_1-----CCG-------------------------------

aV4J_V11_101310_1-----CCTAATCTATGATGC-------------------

aV4J_T14_100710_1-----CTCAC-----------------------------

aV4B_A_419.24_1-------TTTTTGGTGACTCGA-------------------

aV5B_A_419.36_1-------CAC-------------------------------

aV5B_A_419.1_1--------CCCAACG---------------------------

aV5J_V02_090110_1-----CGGTTGAATGCTGGAGGAAGTAAT----------

aV5J_T06_100410_1-----TTAT------------------------------

-------------------------------10--------20--------30---

----------------------....|....|....|....|....|....|....

aV5J_T05_100710_1-----CGCACTGGAGCAGTTCTT----------------

aV5J_V08_091410_1-----CGTCCGGCTCCGCAC-------------------

aV5J_T03_100410_1-----CCAGAGGCATACTGGAGCTTGGACT---------

aV5J_V20_091010_2-----AGACGTGAT-------------------------

aV5m_T08_072712_21----AGATTAAGAAA-----------------------

aV5m_T07_071212_2-----GGGGCTAGGG------------------------

aV5J_T05_100410_1-----AATGGCGT--------------------------

aV5J_T39_100410_1-----CCCGAGCCCACCGAGAGAT---------------

aV6B_A_419.18_1-------CAGCCTGGCCGGT---------------------

aV6J_V11_090110_1-----CCGCGCGGCCGGGTTT------------------

aV6B_A_419.32_1-------CAGCCTCCT-------------------------

aV6m_T05_070912_1-----CAGCCCA---------------------------

aV6m_T11_080912_3-----GTCGAAT---------------------------

aV6B_A_419.23_1-------CCGCCGGGGGTCT---------------------

aV7m_T14B_060712_1----GCCGGCGGG-------------------------

aV7m_T14_071212_1-----CCCCCGGGGGT-----------------------

aV7m_T14_080212_1-----CCGGCC----------------------------

aV7J_V21_090110_1-----TGGACT----------------------------

aV7B_A_419.43_1-------AACCGAGTCA------------------------

aV7m_T20_080212_1-----GGCGAG----------------------------

aV7J_V03_101310_1-----CGGCTTCTGAACTAT-------------------

aV7m_T31_052410_1-----CCTATCGTAT------------------------

aV7m_T16_072712_6-----GATC------------------------------

aV7m_T17_080912_3-----CGCCACCCC-------------------------

aV7J_T15_100710_2-----CCGAT-----------------------------

aV7m_T21_072610_2-----CCCAGTCCGATGTAT-------------------

aV7m_T21_080912_2-----CTG-------------------------------

aV7m_T10_071212_2-----CTCATCG---------------------------

aV7m_T04_051410_2-----CCGTGGGGG-------------------------

aV7J_V04_102810_2-----TGAGAG----------------------------

aV7J_V18_091410_1-----CAATGGGAACTAT---------------------

aV7B_A_419.13_1-------CCGCCGCCTAGGT---------------------

aV7m_T12_072610_1-----TACAATAT--------------------------

aV7m_T16_051410_1-----ATACGGCTGCTGGC--------------------

aV7m_T11B_060712_1----CAGCAGCC--------------------------

aV7m_T20B_060712_1----TAT-------------------------------

aV7m_T18_051410_1-----GGGGAT----------------------------

aV7m_T13B_060712_1----TCGCGAAT--------------------------

aV7J_V02_102810_1-----GGGGCT----------------------------

aV7J_T08_100410_1-----TGGACCCCCTATACT-------------------

aV7m_T12_051410_1-----GGGT------------------------------

aV7J_T04_100710_1-----CGTCCGC---------------------------

aV7J_V03_091410_1-----CTAGTG----------------------------

aV7J_T16_100710_1-----CTATCAGATGTTGGCACC----------------

aV7m_T17_051410_1-----GTCACG----------------------------

aV7J_V04_110210_1-----CGGGT-----------------------------

aV7m_T32_052410_1-----CCTATCCATC------------------------

aV7B_A_419.42_1-------GGCTCTAACT------------------------

aV7J_T07_100410_1-----TTAAGAA---------------------------

-------------------------------10--------20--------30---

----------------------....|....|....|....|....|....|....

aV7J_T20_100410_1-----CTACT-----------------------------

aV7m_T16B_060712_1----AGTCAATAC-------------------------

aV7m_T02_051410_1-----TTGTCGGCTGAT----------------------

aV7J_T01_100710_1-----CCTGGGTAT-------------------------

aV7J_T22_100410_1-----TCTATGAGGCGG----------------------

aV7J_T02_100710_1-----AG--------------------------------

aV7m_T06_051410_1-----GGGATTTC--------------------------

aV7m_T19B_060712_1----GGAG------------------------------

aV7J_V10_091410_1-----CCGGAAT---------------------------

aV1J_V18_090110_1-----CCCGGCGCT-------------------------

aV7m_T20_051410_1-----CTAGGCTCT-------------------------

aV7J_V09_090110_1-----GAGTACATGGAA----------------------

aV9J_V17_091010_1-----TCATCATCATG-----------------------

aV9J_V20_091410_1-----GAAGAGCTGG------------------------

aV9J_T06_100710_1-----TTGGCGTCCCACGCGCCG----------------

aV9J_T09_102810_1-----AGTACCATCGATT---------------------

aV9J_T19_100410_1-----TACT------------------------------

aV9m_T05_071212_1-----GCTGATATAGGAGATAGA----------------

aV9J_V10_090110_1-----CCCCCCCGGAC-----------------------

aV9m_T16_071612_3-----CCCAT-----------------------------

aV9m_T10_080212_3-----GGTGATACT-------------------------

aV9B_A_419.46_2-------AC--------------------------------

aV9J_V05_110210_2-----CAATGGCATTCGGAATGCTGGCACTGAC------

aV9m_T16_072612_1-----TGGGGTCATACT----------------------

aV9J_V12_110210_1-----GAAG------------------------------

aV10.1J_V04_090110_6--AAAT------------------------------

aV10.1m_T01_071212_5--ATCA------------------------------

aV10.1m_T09_072612_5--AATCAGAATGCTAACGCCAAC-------------

aV10.1m_T15_080912_3--TTTCATT---------------------------

aV10.1J_V03_090110_3--TCAAT-----------------------------

aV10.1m_T05_060612_2--AAATCGCT--------------------------

aV10.1m_T17_070612_2--AAACGGCCC-------------------------

aV10.1m_T13_071212_2--AACGAGCAG-------------------------

aV10.1m_T20_062512_1--AGGCTGGA--------------------------

aV10.1J_V13_090110_1--CTC-------------------------------

aV10.1J_V13_091010_1--TTGCTC----------------------------

aV10.1m_T03_062012_1--GTCTGT----------------------------

aV10.1m_T08_060612_1--AAGAAT----------------------------

aV10.1m_T13_062612_1--TCACAC----------------------------

aV10.1m_T16_071212_1--CGGAGAATG-------------------------

aV10.1m_T12_070912_1--AACCAGGATATC----------------------

aV10.1J_V18_102810_1--AGTCGGACTTACGGAGCTGGTACT----------

aV10.1J_T01_100410_1--AAAGTGCCGGTG----------------------

aV10.1J_T46_100410_1--AAACGGGATGATT---------------------

aV10.2J_T23_100410_1--CCATCGTCA-------------------------

aV10.2J_V07_101310_1--CTGAGACATCGCCCCCCTG---------------

aV10.2J_V16_110210_1--ATTGCTC---------------------------

aV10.2J_V22_091410_1--CCTGCTC---------------------------

aV10.1m_T14_062012_1--CGAACGAGAACTATGCAAACAG------------
