# Supplementary material for: Somatic hypermutation of T cell receptor α chain contributes to selection in nurse shark thymus
Source: eLife. 2018 Apr 17;7:e28477. doi: 10.7554/eLife.28477 (PMC5931798; doi:10.7554/eLife.28477)
Supplement: Transparent reporting form [file elife-28477-transrepform.docx]

***eLife’s* transparent reporting form**

We encourage authors to provide detailed information *within their submission* to facilitate the interpretation and replication of experiments. If you have any questions, please contact us: [editorial@elifesciences.org](mailto:editorial@elifesciences.org).

**Sample-size estimation**

- You should state whether an appropriate sample size was computed when the study was being designed
- You should state the statistical method of sample size computation and any required assumptions
- If no explicit power analysis was used, you should describe how you decided what sample (replicate) size (number) to use

Please outline where this information can be found within the submission (e.g., page numbers or figure legends), or explain why this information doesn’t apply to your submission:

We examined the presence or absence of mutation of T cell receptor sequences from sharks. As these data are more descriptive than quantitative, we did not estimate a required sample size.

**Replicates**

- You should report how often each experiment was performed
- You should include a definition of biological versus technical replication
- The data obtained should be provided and sufficient information should be provided to indicate the number of independent biological and/or technical replicates
- If you encountered any outliers, you should describe how these were handled
- Criteria for exclusion/inclusion of data should be clearly stated
- High-throughput sequence data should be uploaded before submission, with a private link for reviewers provided (these are available from both GEO and ArrayExpress)

Please outline where this information can be found within the submission (e.g., page numbers or figure legends), or explain why this information doesn’t apply to your submission:

We present data from a descriptive study of mutation in TcR amplicons. We did not perform an experiment that required replicates for comparison. For T cell receptor alpha chain, we excluded two groups from analysis that did not contain any mutation in the sequenced region. We describe this exclusion in the manuscript on page 11, lines 225-226. There are no high-throughput sequence data. All Sanger sequence data have been deposited in GenBank under the accession numbers given in the manuscript on page 27, lines 568-569 and in figures 1-3, and 5.

**Statistical reporting**

- Statistical analysis methods should be described and justified
- Raw data should be presented in figures whenever informative to do so (typically when N per group is less than 10)
- For each experiment, you should identify the statistical tests used, exact values of N, definitions of center, methods of multiple test correction, and dispersion and precision measures (e.g., mean, median, SD, SEM, confidence intervals; and, for the major substantive results, a measure of effect size (e.g., Pearson's r, Cohen's d)
- Report exact p-values wherever possible alongside the summary statistics and 95% confidence intervals. These should be reported for all key questions and not only when the p-value is less than 0.05.

We used a 1-tailed Student's T-test to analyze mutation frequency by sequence region, predicting that complementarity-determining regions would have more mutation than framework regions (Table 4; Fig 6). We used chi-square tests to compare the number of mutations in hotspots (Table 5) and to G and C residues by sequence region (Table 4). Significance was set at p=0.05. Statistical tests are explained in the methods section for each analysis.

Please outline where this information can be found within the submission (e.g., page numbers or figure legends), or explain why this information doesn’t apply to your submission:

(For large datasets, or papers with a very large number of statistical tests, you may upload a single table file with tests, Ns, etc., with reference to page numbers in the manuscript.)

**Additional data files (“source data”)**

- We encourage you to upload relevant additional data files, such as numerical data that are represented as a graph in a figure, or as a summary table
- Where provided, these should be in the most useful format, and they can be uploaded as “Source data” files linked to a main figure or table
- Include model definition files including the full list of parameters used
- Include code used for data analysis (e.g., R, MatLab)
- Avoid stating that data files are “available upon request”

Please indicate the figures or tables for which source data files have been provided:

All source data are found in existing tables. Data for Fig 6 can be found in Table 1 as well as in "Figure 6 - Source Data."
